# MicroRNA-382 induced by HIF-1α is an angiogenic miR targeting the tumor suppressor phosphatase and tensin homolog

**DOI:** 10.1093/nar/gku515

**Published:** 2014-06-09

**Authors:** Jin-Kyung Seok, Sun Hee Lee, Min Jung Kim, You-Mie Lee

**Affiliations:** 1 Research Institute of Pharmaceutical Sciences, College of Pharmacy, 80 Daehak-ro, Buk-gu, Daegu, 702-701, Republic of Korea; 2 School of Life Sciences and Biotechnology, Kyungpook National University, 80 Daehak-ro, Buk-gu, Daegu, 702-701, Republic of Korea

## Abstract

Recent studies have revealed that microRNAs (miRs) play important roles in the regulation of angiogenesis. In this study, we have characterized miR-382 upregulation by hypoxia and the functional relevance of miR-382 in tumor angiogenesis. miRs induced by hypoxia in MKN1 human gastric cancer cells were investigated using miRNA microarrays. We selected miR-382 and found that the expression of miR-382 was regulated by HIF-1α. Conditioned media (CM) from MKN1 cells transfected with a miR-382 inhibitor (antagomiR-382) under hypoxic conditions significantly decreased vascular endothelial cell (EC) proliferation, migration and tube formation. Algorithmic programs (Target Scan, miRanda and cbio) predicted that phosphatase and tensin homolog (PTEN) is a target gene of miR-382. Deletion of miR382-binding sequences in the PTEN mRNA 3′-untranslated region (UTR) diminished the luciferase reporter activity. Subsequent study showed that the overexpression of miR-382 or antagomiR-382 down- or upregulated PTEN and its downstream target AKT/mTOR signaling pathway, indicating that PTEN is a functional target gene of miR-382. In addition, PTEN inhibited miR-382-induced *in vitro* and *in vivo* angiogenesis as well as VEGF secretion, and the inhibition of miR-382 expression reduced xenograft tumor growth and microvessel density in tumors. Taken together, these results suggest that miR-382 induced by hypoxia promotes angiogenesis and acts as an angiogenic oncogene by repressing PTEN.

## INTRODUCTION

Hypoxia, a reduction in tissue oxygen tension, is associated with various pathophysiological events. In particular, hypoxia activates genetic programs that facilitate cellular adaptations by changing cell signaling and gene expression to promote cell survival, migration, angiogenesis, cancer cell invasion, metastasis and resistance to chemotherapy (1). Hypoxia effectively drives angiogenesis, which represents a compensatory mechanism against tissue ischemia (2,3) and hypoxia-induced angiogenesis is mainly mediated by vascular endothelial growth factor (VEGF) via the activation of hypoxia inducible factor (HIF) (4,5). Because of their hypoxic nature, virtually all solid tumors express high levels of VEGF, which contributes to the high degree of leakiness and tortuosity of tumor vasculature (6).

Micro ribonucleic acids (miRNAs) are small non-coding RNAs that participate in post-transcriptional gene regulation in plants and humans (7,8). About 5300 human genes are suspected targets of miRNAs, which recognize messenger RNAs (mRNAs) exhibiting sequence complementarity and act on them to inhibit protein translation or cleavage of target mRNAs, by base-pairing with their 3′-untranslated regions (3′-UTR) (9). Approximately 1% of the human genome is dedicated to miRNAs, which in turn regulate roughly one-third of all mRNAs in the human body. Since the discovery of the first miRNA in 1993 (10), over 10 000 miRNA genes have been documented in the ‘MicroRNA Registry’ (11). Therefore, miRNAs are thought to regulate the expression of most genes and consequently to play critical roles in the coordination of a wide variety of processes, including differentiation, proliferation, angiogenesis, death and metabolism (12–15).

Aberrant miRNA expression is associated with a number of human diseases (14) and the identification of angiogenesis-regulatory miRNAs has opened a new avenue to the treatment of vascular diseases. Mimics of pro-angiogenic miRNAs or antagomiRs of anti-angiogenic miRNAs can be used to increase angiogenesis in pathological settings of insufficient angiogenesis, such as, in myocardial infarction (MI) and ischaemia. Conversely, mimics of anti-angiogenic miRNAs or antagomiRs of pro-angiogenic miRNAs may be effective in settings of pathological vascularization, such as in tumor angiogenesis or retinopathy (16). However, little is known about the role played by miRNAs during the angiogenic response to hypoxia.

Phosphatase and tensin homolog (PTEN) is a tumor suppressor in various cancers, but the relation between PTEN and angiogenesis has not been well documented. In this study, we investigated the roles of miRNA-382, a hypoxia- and HIF-1α-induced miRNA, in angiogenesis. The upregulated or downregulation of miR-382 significantly increased or decreased endothelial cell proliferation, migration and tube formation, suggesting that miR-382 is an angiogenic miR. In addition, algorithmic programs predicted that PTEN is targeted by miR-382, and *in vitro* and *in vivo* angiogenesis assays showed that PTEN is involved in miR-382-regulated angiogenesis under hypoxic conditions. Furthermore, the decreased growth of xenografted human gastric cancer overexpressing antagomiR-382 was attributed to low microvessel density. This study shows that miR-382 modulates angiogenesis in highly proliferative tumors and under ischemic conditions by targeting PTEN.

## MATERIALS AND METHODS

### Ethics statement

Animal care and experimentation were performed in accordance with procedures approved by the KNU (Kyngpook National University) Animal Care and Use Committee.

### Cell culture and hypoxia

MKN1 human gastric cancer cells (Korean Cell Line Bank, Seoul, South Korea) were maintained in RPMI-1640 (Hyclone, Logan, UT, USA), and bovine aortic endothelial cells (BAECs, passage 3–7) were maintained in Dulbecco's Modified Eagles Medium (DMEM) (Hyclone), containing 10% fetal bovine serum (FBS) (Hyclone) and 1% antibiotics. For hypoxia, the cells were incubated at 5% CO_2_, 1% O_2_, 94% N_2_ in a hypoxic chamber (ASTEC, Fukuoka, Japan) at 37°C.

### Target prediction for microRNAs

Bioinformatics prediction of target genes and miRNA binding sites was performed using different databases: TargetScan (http://www.targetscan.org/), miRanda (http://www.microrna.org/) and cbio (http://cbio.mskcc.org/mirnaviewer/). An overlapping target molecule for microRNA382 from these databases was PTEN and was considered for further experimental analysis.

### RNA extraction and microarray analysis

Total RNA from MKN1 cells treated with hypoxia for 24 h was prepared using Trizol Reagent (Invitrogen, Carlsbad, CA, USA). A human miRNA array (GenoSensor, Tempe, AZ, USA) was used to compare miRNA expression in MKN1 gastric cancer cells under normoxic and hypoxic conditions. Total RNA (10 ng) was labeled with biotin and hybridized to the miRNA array according to the MAUI hybridisation system. Acquired images and the signal intensities of miRNA spots were analyzed using GeneSpring GX (Agilent Technologies, Santa Clara, CA, USA) and normalized based on global signal intensity according to the manufacturer's instructions. Differential miRNA expression was determined using a two-sided Student's *t*-test on a single miRNA basis; significance was accepted for changes >2-fold. Our microarray data were deposited in a data base (Gene Expression Omnibus, file number: GSE 56870).

### Quantitative real-time reverse transcription polymerase chain reaction (qReal-Time PCR)

Total RNA was extracted from the cells using TRIzol Reagent and a Purelink miRNA Isolation Kit (Invitrogen), according to the manufacturer's instructions. Complementary DNA (cDNA) was generated from 500 ng of total RNA using the GenoExplorer™ miRNA First-Strand cDNA Core Kit (GenoSensor). Real-time PCR (Applied Biosystems 7300, Carlsbad, CA, USA) was performed using SYBR Green PCR Master Mix (Qiagen, Valencia, CA, USA) and GenoExplorer™ miRNA qPCR primer sets for each microRNAs (Genosensor) or miScript miR-382 primer assay kit (Qiagen). The relative levels of mature miRNAs in MKN1 cells were calculated with respect to U6 RNA (internal control). For semi-quantitative RT-PCR, the following primers were used: PTEN, forward 5′- GGACGAACTGGTGTAATGAT-3′ and reverse 5′-TCTACTGTTGTGAAGTACAGC-3′ and β-actin, forward 5′-GATCCACATCTGCTGGAA-3′ and reverse 5′-GACTACCTCATGAAGATC-3′.

### Transfection of miR- or antagomiR-382 and conditioned media collection

To overexpress or inhibit miRNAs, miRNA mimics (Qiagen #MSY0000737) or antago-miR (Qiagen #MIN0000737) were transfected using Hiperfect transfection reagent (Qiagen), according to the manufacturer's instructions. After cloning the mature miR-382 into pENTR™/H1/TO vector (Invitrogen), MKN1 cells were transfected and conditioned media (CM) was obtained by culturing cells in serum free media for 24 h.

### Cell proliferation assay

BAECs (5 × 10^3^ cells/well) were seeded onto 48-well plates and allowed to attach for 24 h. The medium was then replaced with low serum medium (1% FBS in DMEM) for 20 h and treated with CM from MKN1 cells transfected with miR-382-cloned pENTR™/H1/TO vector or PNA™-antagomiR-382. Cells were then treated with 1 μCi/well of [^3^H]-thymidine and incubated for an extra 4 h. After fixation, cells were solubilized with 0.3N sodium hydroxide. Cell-associated radioactivity was measured using a liquid scintillation counter (Perkin Elmer, Waltham, MA, USA).

### *In vitro* migration assay

Migration assays were performed using 8 μm polycarbonate filters in 24-transwell plates (Costar, Lowel, MA, USA). The lower sides of the filters were coated with 10 μl of type I collagen (0.5 mg/ml). CM obtained from transfected MKN1 cells mentioned above was loaded into the lower chamber and 4 × 10^4^ BAECs were seeded into the upper chamber in serum-free media and cultured for 20 h. Cells were then fixed with methanol and stained with hematoxylin (Sigma, St Louis, MO, USA) and eosin (Sigma). Cells on the upper filter surface were removed and migration was determined by counting cells that had migrated to the lower filter side under a microscope at 200×. Samples were assayed twice in triplicate.

### Tube formation assay

BAECs were seeded on 48-well culture plates coated with Matrigel (BD Biosciences, San Diego, CA, USA). CM from transfected MKN1 cells mentioned above was added and incubated for 12 h under normoxic conditions. Morphologic changes were observed under a microscope and photographed at 100×.

### Chorioallantoic membrane (CAM) assay

CM from transfected MKN1 cells or phorbol 12-myristate-13-acetate (PMA) was mixed with Matrigel (1:1) and loaded onto CAM of chick embryos 3.5 days after fertilization. Four days after loading the matrigel, the solid matrigel was detached from CAM and microvessels were observed in the matrigel under a microscope (Olympus, Japan, 40×). The total length of microvessels in matrigel was determined using photographs. Fifteen eggs per group were used in each experiment, which were performed in triplicate.

### Whole cell extract preparation and western blotting

MKN1 cells transfected with miR-382 cloned plasmid (pENTR™/H1/TO vector) or PNA™ miR-382 inhibitor (antagomiR-382) and cultured under hypoxic or normoxic conditions were harvested with a lysis buffer (iNtRon Biotech, South Korea), and equal amounts of protein were subjected to SDS-PAGE. Proteins were transferred to nitrocellulose membranes (Whatman, Maidstone, UK), and membranes were blocked with 5% non-fat skim milk in Tris-buffered saline (TBS) containing 0.1% Tween-20 for 30 min at room temperature (RT). After blocking, membranes were incubated with specific antibodies against PTEN (Cell Signaling, Danver, MA, USA) or β-actin (Santa Cruz Biotechnology, Santa Cruz, CA, USA) overnight at 4°C followed by horseradish peroxidase-conjugated mouse- or rabbit-IgG at RT, and then developed with West Pico chemiluminescent substrate (Pierce, Woburn, MA, USA). Relative protein expression levels (plotted below the band pictures) were quantified using ImageJ (NIH, Bethesda, MD, USA) and normalized against internal controls and a graph was produced. Three independent experiments were performed, and representative results are shown.

### Cloning and the dual luciferase assay

Two 3′-UTR regions of PTEN mRNA (369 or 244 bp) matching miR-382 sequences were synthesized by PCR and cloned into pGL3 control vector downstream of the luciferase open reading frame (ORF) after digestion with XbaI. The primers were: for #21, forward 5′-GCCGTGTAATTCTAGAAGAGGAGCCGTCAAATCCAG-3′ and reverse 5′-CCGCCCCGACTCTAGAGCAAGTGTCAAAACCCTGTGGA-3′ and for #956, forward 5′-GCCGTGTAATTCTAGAGTGGAGGCTATCAACAAAG-3′ and reverse 5′ CCGCCCCGACTCTAGACACATGAAGCATCCACA-3′. Deletion mutants of matching sequences for PTEN mRNA 3′-UTR region cloned in pGL3 control vector were generated by and purchased from Bioneer (Daejeon, South Korea). The promoter region of miR-382 (−4 kb) was synthesized and cloned into pGL3 vector upstream of the luciferase ORF. MKN1 cells were cotransfected with cloned pGL3 vector and renilla luciferase vector. Luciferase assays were performed using the Dual-Glo Luciferase^®^ Reporter Assay System (Promega, Madison, WI, USA), according to the manufacturer's instructions.

### Enzyme-linked immunosorbent assay (ELISA)

Amounts of VEGF protein secreted by MKN1 cells into the medium were determined using the VEGF ELISA kit (R&D Systems, Wiesbaden, Germany). Cells were plated in six-well plates, cultured until 80–90% confluent, and transfected with miR-382 oligomers. After 24 h, the medium was replaced and amounts of VEGF secreted into the medium were quantified.

### Animal experiment

Five-week-old athymic nude mice [BALB/cSIc-nu (18–21 g)] were purchased from the Institute of Medical Science, University of Tokyo (Japan) and maintained under specific pathogen-free conditions on a standard diet. Nude mice were inoculated with MKN1 cells (1 × 10^6^) subcutaneous (s.c.) and treated with echinomycin (0.12 mg/kg body weight) every 2 days for 16 days.

Stable cell lines were established by transfecting MKN 1 cells with antagomiR-382-cloned-pEZX vector or control-pEZX vector, and then selected in the presence of puromycin. Cells (5 × 10^6^) stably expressing antagomiR-382 or control miR were injected s.c. into the flanks of 6-week-old nude mice and tumor sizes were measured daily for 30 days. Tumor volumes (cm^3^) were calculated by multiplying height by length by depth.

### Immunohistochemistry (IHC)

Serial sections (5 μm) of tumor tissue were mounted on poly-l-lysine-coated slides. Each section was immunostained with antibodies against CD31 (BD Biosciences) and HIF-1α (BD Biosciences) and visualized by incubation with the appropriate biotin-conjugated secondary antibodies and immmunoperoxidase detection using the Vectastain ABC Elite kit (Linaris, Werthein, Germany) and diaminobenzidine (DAB) substrate (Vector Laboratory, UK). Counterstaining was performed with hematoxylin. The immunoreactions were observed under an optical microscope at 200×.

### Statistical analysis

ANOVA was used to assess the significance of intergroup differences. Statistical significance was accepted for *P*-values < 0.05, and results are represented as means ± standard deviations (SD).

## RESULTS

### Differential expression of microRNAs under normoxic and hypoxic conditions

**Figure 1.**
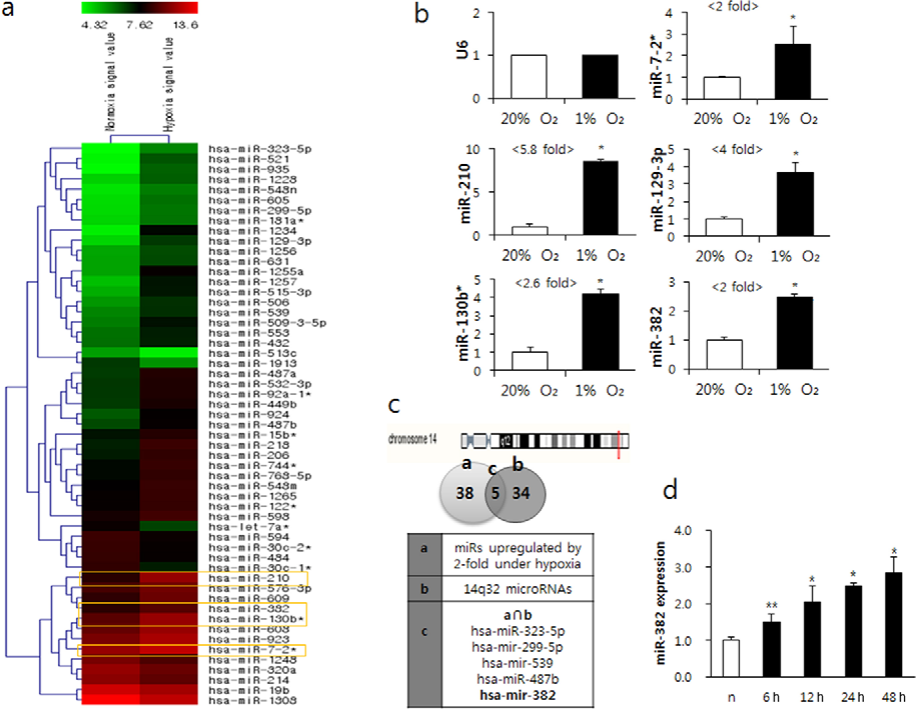
MicroRNAs differentially expressed in normoxia and hypoxia. (**a**) The expression of microRNAs was investigated using a microRNA microarray and total RNA isolated from MKN1 cells exposed or not to hypoxia (1% O_2_) for 24 h. The heatmap shows the differential expression patterns of microRNAs under normoxic and hypoxic conditions. Green means low expression, red means high expression, and darker reds substantially higher expression. (**b**) Quantitative real-time RT-PCR was performed to confirm the expression patterns of the selected miRs, miR-7-2*, miR-210, miR-129-3p, miR-130b* and miR-382. Relative miR expression was normalized against RN-U6. (**c**) Location of the chromosome 14q32 microRNA cluster and a Venn diagram showing overlapping miRNAs between miRs upregulated at >2-fold under hypoxia in the microarray and 14q32 miRs. (**d**) Time-dependent expressions of miR-382 after exposure to hypoxia (H) for 6, 12, 24 or 48 h. Significance, *P < 0.05 versus the normoxic (20% O_2_, *n*) control.

To investigate the miRNAs involved in angiogenesis under hypoxic conditions, we used a miRNA microchip. Forty-three miRNA probes were found to be upregulated by over 2-fold, while 12 miRNA probes were downregulated by over 2-fold under hypoxic conditions (Figure 1a). When we confirmed the microchip data using quantitative real-time PCR for miR-7-2*, -210, -130b*, -129-3p, and -382, these miRNAs were found to be significantly upregulated by 24 h of hypoxia (Figure 1b). miR-210 has already been reported to be involved in various cellular responses in hypoxic cells and a pleiotrophic hypoxamir (17). miR-130b* has been reported to target colony-stimulating factor (CSF)-1 and to be involved in multidrug resistance in ovarian cancer (18) and in papillary thyroid carcinoma development (19). However, the roles of miR-382 have not been determined in relation to hypoxia, angiogenesis or cancer progression. Microarray data showed that its cluster at chromosome 14 (14q32 miRNAs) was upregulated under hypoxia, and we found that miR-382 overlapped between the upregulated miRNAs under hypoxia and the 14q32 miRNAs increased by 2-fold (Figure 1c). Therefore, we selected miR-382 as a candidate miR in angiogenic function for further study. When MKN1 cells were exposed to hypoxia for 6, 24 or 48 h, the expression of miR-382 was increased time-dependently until 48 h (Figure 1d), suggesting that its expression is substantially upregulated by subchronic hypoxia.

### HIF-1α was involved in miR-382 expression

**Figure 2.**
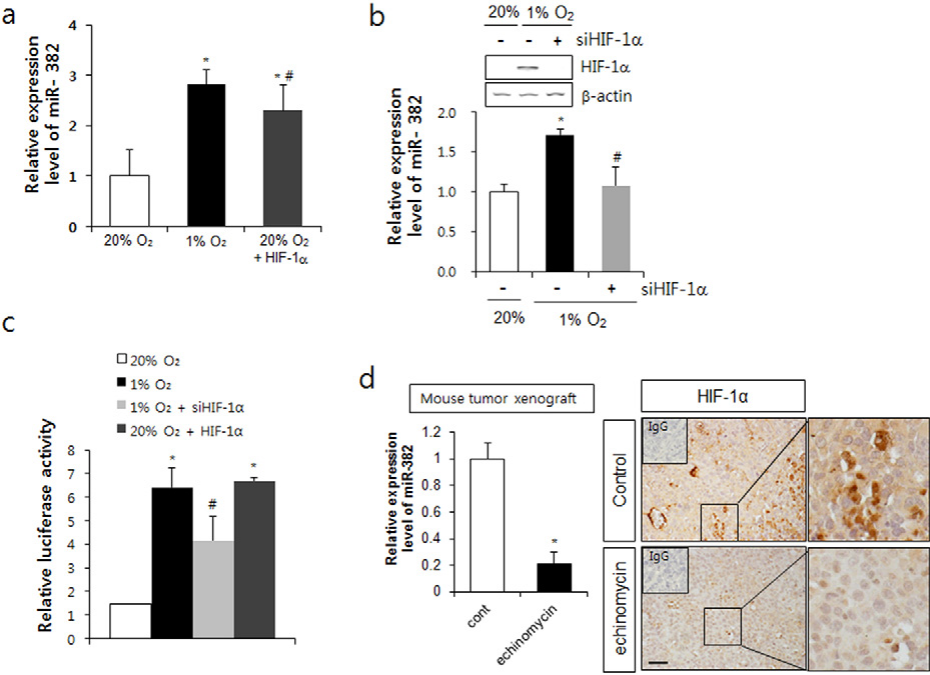
miR-382 expression was regulated by HIF-1α. (**a**) MKN1 cells were transfected with pEGFP-HIF-1α or exposed to hypoxia for 24 h, and relative miR-382 expression levels were determined by real-time qPCR. (**b**) After transfection with the HIF-1α siRNA (siHIF-1α), cells were exposed to hypoxia for 24 h and HIF-1α mRNA levels were determined by RT-PCR. Relative miR-382 expression levels were also confirmed by real-time qPCR. (**c**) MKN1 cells were transfected with miR-382 promoter reporter plasmid and pEGFP-HIF-1α or siHIF-1α, and relative luciferase activities were determined. (**d**) Nude mice were inoculated with MKN1 cells (1 × 10^6^) s.c., treated with echinomycin (0.12 mg/kg body weight) every 2 days for 16 days and expression of miR382 and HIF-1α in tumor masses were measured by real-time PCR and immunohistochemistry, respectively. Pictures from IHC were shown in right panel and magnified images of the boxed regions are shown right in each picture. Upper left small box in IHC picture shows IgG control staining. Scale bar = 100 μm. Three independent experiments were performed in triplicate.*P < 0.05 versus normoxic control. ^#^*P* < 0.05 versus hypoxic control.

We investigated the involvement of HIF-1α in miR-382 expression under hypoxia. Overexpression of HIF-1α (pEGFP-HIF-1α) upregulated endogenous miR-382 expression (Figure 2a), whereas HIF-1α siRNA downregulated miR-382 levels, under hypoxic conditions (Figure 2b). To further examine the regulation of miR-382 by HIF-1α under hypoxic conditions, we cloned the miR-382 promoter region (−4 kb) harboring two hypoxia responsive element (HRE) sequences into a luciferase reporter vector. The luciferase activity of miR-382 promoter was increased by hypoxia in MKN1 cells (Figure 2c). When MKN1 cells were transfected with pEGFP-HIF-1α, miR382-reporter activity was increased to the level of the hypoxia control. However, transfection with the HIF-1α siRNA diminished hypoxia-induced miR-382 reporter activity (Figure 2c). When we treated gastric cancer xenografted nude mice with a HIF-1α-specific inhibitor, echinomycin, for 10 days, echinomycin decreased the expression of miR-382 by 0.2-fold in tumor tissues, confirmed by real-time PCR, indicating that HIF-1α is involved in miR-382 expression in tumor tissues. In echinomycin-treated tumor tissues, HIF-1α expression was decreased (Figure 2d). These results show that HIF-1α is essential for the regulation of miR-382 expression.

### Controlled expression of miR-382 modulated *in vitro* angiogenesis

**Figure 3.**
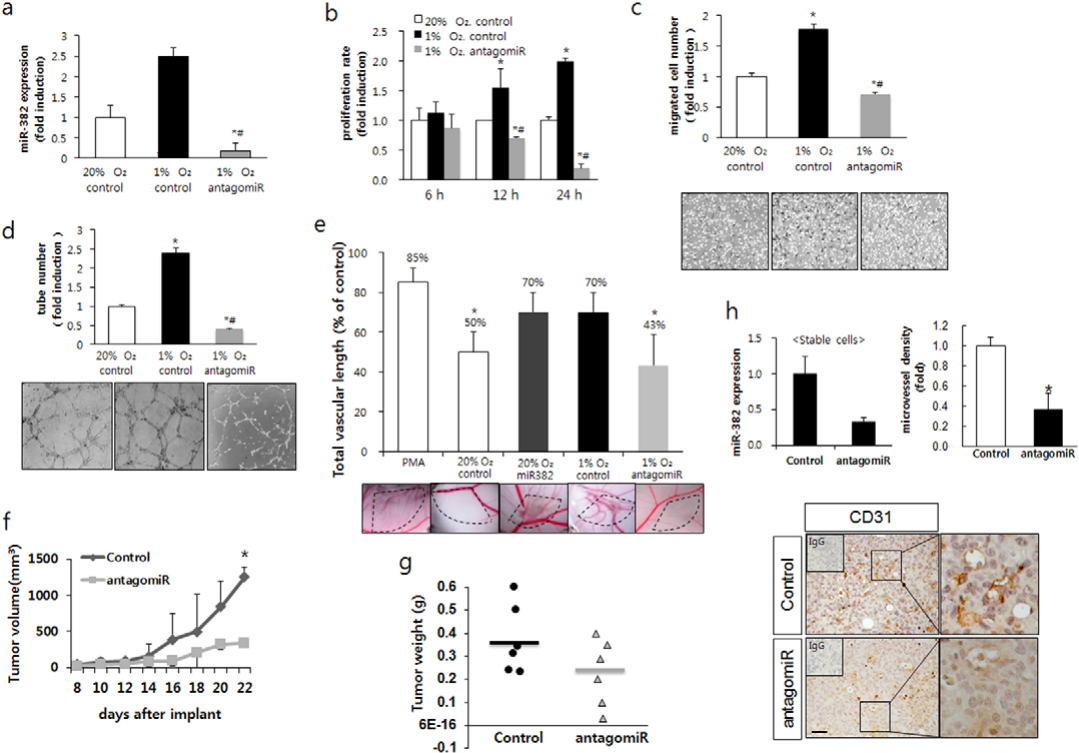
Inhibition of miR-382 suppressed hypoxia-induced angiogenesis. (**a**) MKN1 cells were transfected with synthesized antagomir-382 oligomers (70 nM) and the expression of miR-382 was confirmed by real-time RT-PCR. (**b**) Using conditioned media (CM) collected from the transfected MKN1 cells described in (a), proliferation assays were performed with BAECs exposed to normoxic (20% O_2_) or hypoxic (1% O_2_) conditions for 6, 12 or 24 h. (**c**) Boyden chamber migration assays were performed with BAECs treated with the same CM used in (b). Cells that migrated through the membrane (8 μM pore size) were stained and counted. (**d**) Tube formation assays were performed using BAECs treated with the CM used in (b). Completed tube numbers were counted and plotted. (**e**) CM obtained from miR-382 or antagomiR-382 transfected MKN1 cells under normoxic (20% O_2_) or hypoxic (1% O_2_) conditions were administered to 6.5-day-chick embryo CAM and new blood vessel formation was observed 4 days later. Eggs showing new vessel formation (positive) are expressed as percentages of the number of eggs tested (noted above the bars). Fifteen eggs were tested per experiment and experiments were performed in triplicate. (**f**) Xenografted nude mice were injected subcutaneously with MKN1 cells stably expressing control-pEZX or antagomiR-pEZX vector, and observed for 22 days. From one week post-injection, tumor volumes (length × width × height, mm) were measured on alternate days; animals were sacrificed 29 days post-injection. (**g**) The graph shows tumor weights measured at day 29. (**h**) Immunohistochemical analysis against CD31 in tumor masses was performed and microvessel density for CD31 positive cells was counted and graphed (right panel). Left panel shows miR-382 expression level in antagomiR-382-stable transfectants. Pictures from IHC were shown in lower panel and magnified images of the boxed regions are shown right each picture. Upper left small box in IHC picture shows IgG control staining. Scale bar = 100 μm. Significance: *P < 0.05 versus the control tumor (or 20% O_2_ normoxic group) and ^#^*P* < 0.05 versus the hypoxic control (1% O_2_).

Next, we tested whether the inhibition of miR-382 blocked hypoxia-induced angiogenesis. AntagomiR-382 successfully inhibited miR-382 expression hypoxic conditions (Figure 3a). In the *in vitro* angiogenesis assay with CM, antagomiR-382 suppressed the hypoxia-induced proliferation of BAECs after 12 and 24 h of hypoxia (Figure 3b). In the transwell migration assay, hypoxia significantly increased endothelial cell (EC) migration but antagomiR-382 significantly inhibited hypoxia-induced migration (Figure 3c). In addition, we checked the ability of ECs to form capillary-like structures on matrigel. Hypoxia significantly increased the tubulogenesis of ECs, whereas tube networks were dramatically suppressed by antagomiR-382, as evidenced by incomplete sprouting, branching and defective network formation between tubes (Figure 3d). Next, we confirmed these *in vitro* results by performing *in vivo* CAM assays using CM from miR-382- or antagomiR-382-transfected MKN1 cells. miR-382 overexpression and hypoxia significantly increased angiogenesis in matrigel inoculated under the CAM of chick embryos, whereas antagomiR-382 and hypoxia significantly decreased vessel formation (Figure 3e). These results suggest that the inhibition of miR-382 can block hypoxia-induced angiogenesis *in vitro* and *in vivo*.

To determine whether miR-382-induced angiogenesis was associated with tumor growth, we prepared MKN1 gastric cancer cells stably expressing antagomiR-382. Xenograft tumor masses made of stable cells expressing antagomiR-382 grew slower than tumors made of mock vector expressing cells. Furthermore, the tumor weights and growth rates of antagomiR-382-expressing tumor cells were significantly inhibited as compared with those of mock transfected cells (Figure 3f and g). Immunohistochemistry confirmed that CD31-expressing microvessel density was inhibited in antagomiR-382-expressing tumor cells (Figure 3h).

### PTEN is a target of miR-382

To identify the molecular target of miR-382, we predicted target candidates using three algorithmic programs (TargetScan, miRanda and cbio) and by examining miR binding efficacy to the 3′-UTR regions of target genes (Supplemental Figure S1). PTEN was identified by all three programs, and therefore, was as a candidate target of miR-382. It was previously reported that PTEN inactivation increases angiogenesis in gastric cancer patients via VEGF (20), and that PTEN is a tumor suppressor frequently mutated or deleted in human cancer (21).

**Figure 4.**
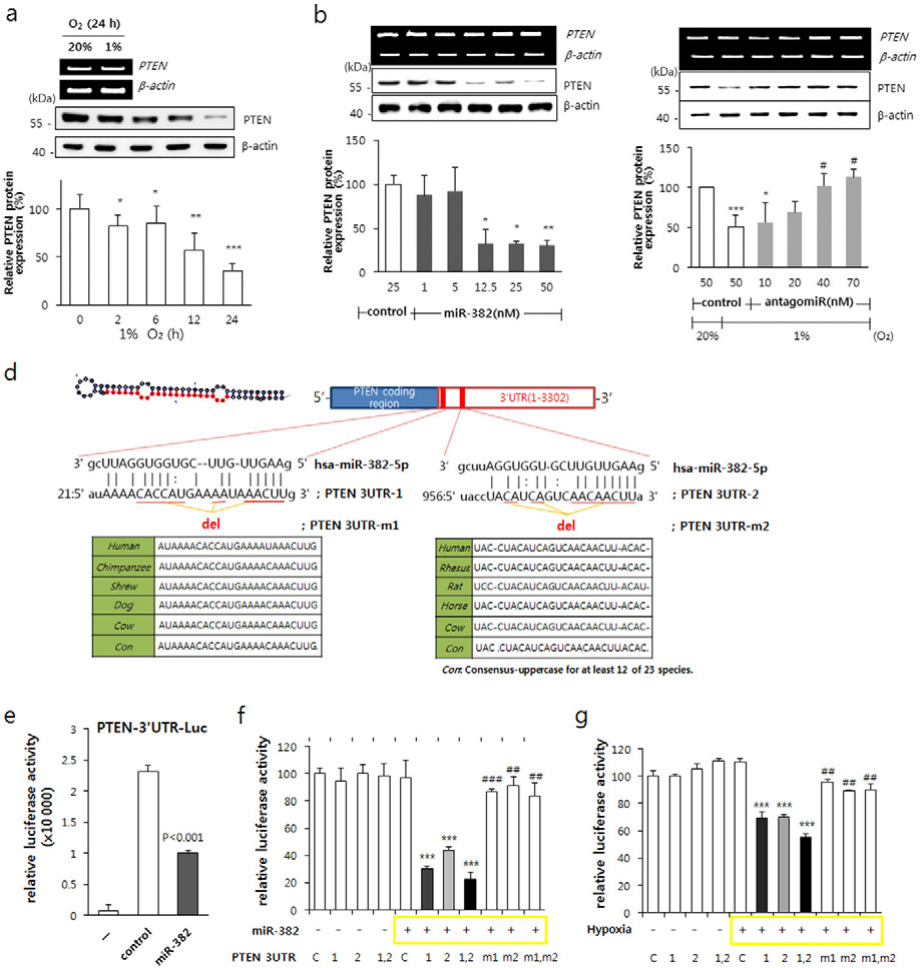
Identification of PTEN as a target of miR-382. (**a**) PTEN expression was determined by western blot analysis after MKN1 cells were exposed to hypoxia for 2, 6, 12 and 24 h. β-actin was used as an internal control. (**b**) MKN1 cells were transfected with miR-382 oligomers (1, 5, 12.5, 25 and 50 nM) and PTEN expression was determined using β-actin as an internal control. (**c**) MKN1 cells were transfected with antagomiR-382 oligomers (10, 20, 40 and 70 nM) and exposed to hypoxia. PTEN expression was determined by western blotting using β-actin as an internal control. (**d**) Hairpin structure of premiR-382 is shown in the upper left. Sequence alignment of miR-382 with the PTEN 3′-UTR region is shown in the middle box. Comparison of miR-382 sequences in human, rhesus monkey, rat, horse and cow. Con means consensus-uppercase for at least 12 of 23 species. (**e**) MKN1 cells were transfected with pGL3 control vectors cloned downstream of the luciferase ORF harboring two PTEN mRNA 3′-UTR regions matching the miR-382 sequences in the presence or absence of miR-382 oligomers. Promoter activity was determined at 24 h after transfection as described in ‘Materials and Methods’ section. (**f**) The transfection procedures used were as described in (e) with two types of PTEN mRNA 3′-UTR regions matching miR-382 sequences, or mutants of the PTEN mRNA 3′-UTR regions, or their combinations in the presence or absence of miR-382 oligomers. Promoter activities were determined at 24 h after transfection as described in ‘Materials and Methods’ section. (**g**) Transfection was performed as described in (e) and followed by exposure to hypoxia for 24 h. Promoter activities were determined as described in ‘Materials and Methods’ section. Three independent experiments were performed in triplicate. *P < 0.05, **P < 0.01 or ***P < 0.001 versus 20% O_2_ or normoxic control. ^#^*P* < 0.05, ^##^*P* < 0.01 or ^###^*P* < 0.001 versus hypoxic control.

To determine whether PTEN was downregulated under hypoxic conditions, MKN1 cells were exposed to hypoxia for 24 h and the mRNA and protein levels of PTEN were examined. PTEN was significantly suppressed in a time-dependent manner and completely disappeared at 24 h under hypoxic conditions. However, PTEN mRNA levels were unchanged after 24 h of hypoxia compared to normoxic conditions (Figure 4a). In addition, the overexpression of miR-382 dose-dependently decreased PTEN protein levels but not its mRNA expression (Figure 4b). Additionally, dose-dependent inhibition of miR-382 by antagomiR recovered PTEN expression under hypoxic conditions (Figure 4c). These results suggest that PTEN is regulated by miR-382 and that it might be a target of miR-382.

We next examined whether PTEN was a real target of miR-382. When we aligned the sequences of miR-382 with PTEN 3′-UTR sequences in various species, a highly conserved 3′-UTR region of the PTEN gene was found to match the miR-382 sequence (Figure 4d). Thus, we cloned two 3′-UTR regions of the PTEN mRNA matching the miR-382 sequences into pGL3 control vector downstream of the luciferase ORF. One harbored a 25-bp miR-382-matching sequence (starting from 21, ‘3UTR-1’) and the other, a downstream 23-bp sequence (starting from 956, ‘3UTR-2’) of the 3′-UTR region of PTEN mRNA. We found that MKN1 cells overexpressing miR-382 oligomers exhibited significantly less luciferase activity than cells transfected with control miR (Figure 4e), suggesting that PTEN expression was regulated by miR-382. To confirm this result, we prepared deletion mutants of miR-382-matching sequences in PTEN mRNA 3′-UTR regions (3UTR-m1 and 3UTR-m2, in Figure 4d). Mutants of these two regions in PTEN mRNA 3′-UTR did not inhibit the reporter activity induced by miR-382 oligomers (Figure 4f), suggesting that miR382 targets PTEN translation. Under hypoxic conditions, the luciferase reporter activities of 3UTR-1, 3UTR-2 or both were decreased, but those of the deletion mutants (3UTR-m1 and 3UTR-m2) were resistant to the hypoxic repression of PTEN mRNA reporter activity (Figure 4g). These results confirmed that PTEN is a real target of miR-382.

### miR-382 activated the AKT/mTOR signaling pathway

PTEN negatively regulates the phosphoinositide 3-kinase (PI3K) signaling pathway by dephosphorylating the 3′ position of phosphatidylinositol-3,4,5-triphosphate (PIP3) (22). The PI3K/AKT/mTOR pathway is involved in intracellular signaling for cell growth, proliferation and survival (23). Therefore, the suppression of PTEN function by mutation or epigenetic regulation leads to tumorigenic susceptibility and progression (20,21).

**Figure 5.**
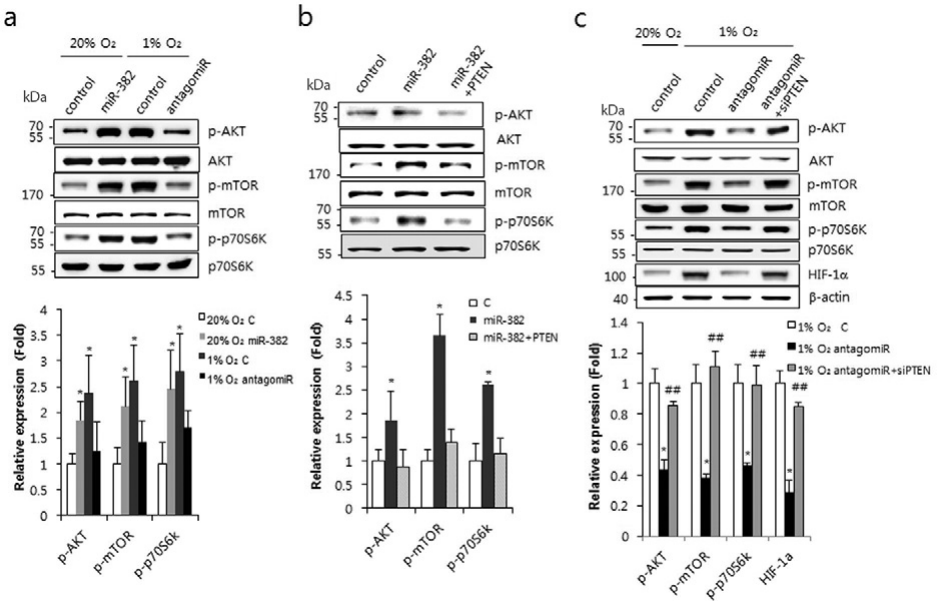
miR-382 activated the AKT/mTOR signaling pathway. (**a**) MKN1 cells were transfected with miR-382 (50 nM) or with antagomiR-382 (70 nM) and exposed to normoxic or hypoxic conditions. Western blot analysis was performed using antibodies against the phosphorylated or native forms of AKT, mTOR and p70S6K. (**b**) MKN1 cells were transfected with PTEN full-length plasmid and/or miR-382 oligomers and western blot analysis was performed at 24 h after transfection. Expression of the phosphorylated or native forms of AKT, mTOR and p70S6K was determined using appropriate antibodies. (**c**) MKN1 cells were transfected with siRNA PTEN and/or antagomiR-382 (70 nM) and exposed to normoxic or hypoxic conditions. Western blot analysis was performed using antibodies against HIF-1α and the phosphorylated or native forms of AKT, mTOR and p70S6K. Relative protein expression (phosphorylated/unphosphorylated protein) was calculated and plotted. β-actin was used for internal control for HIF-1α in (c). Three independent experiments were performed in triplicate. *P < 0.05, **P < 0.01 or ***P < 0.001 versus 20% O_2_ or normoxic control. ^##^*P* < 0.01 versus hypoxic control.

As PTEN is an inhibitor of the PI3K/AKT/mTOR signaling pathway and hypoxia increases miR-382, if PTEN is a real target of miR-382, the PI3K/AKT/mTOR signaling pathway might be increased by hypoxia or miR-382 synthetic oligomers. miR-382 or hypoxia activated AKT, mTOR and p70S6K. However, the inhibition of miR-382 by antagomiR-382, under hypoxic conditions, restored the activities of AKT, mTOR and p70S6K (Figure 5a). When PTEN was co-expressed with miR-382, the miR-382-induced activities of AKT, mTOR and p70S6K were significantly inhibited (Figure 5b). Furthermore, silencing PTEN with siRNA recovered the antagomiR-382-induced inhibition of AKT, mTOR and p70S6K activities under hypoxic conditions (Figure 5c), suggesting that miR-382 regulates the AKT/mTOR signaling pathway by targeting PTEN under hypoxic conditions.

### PTEN inhibited miR382-induced angiogenesis mediated by VEGF

**Figure 6.**
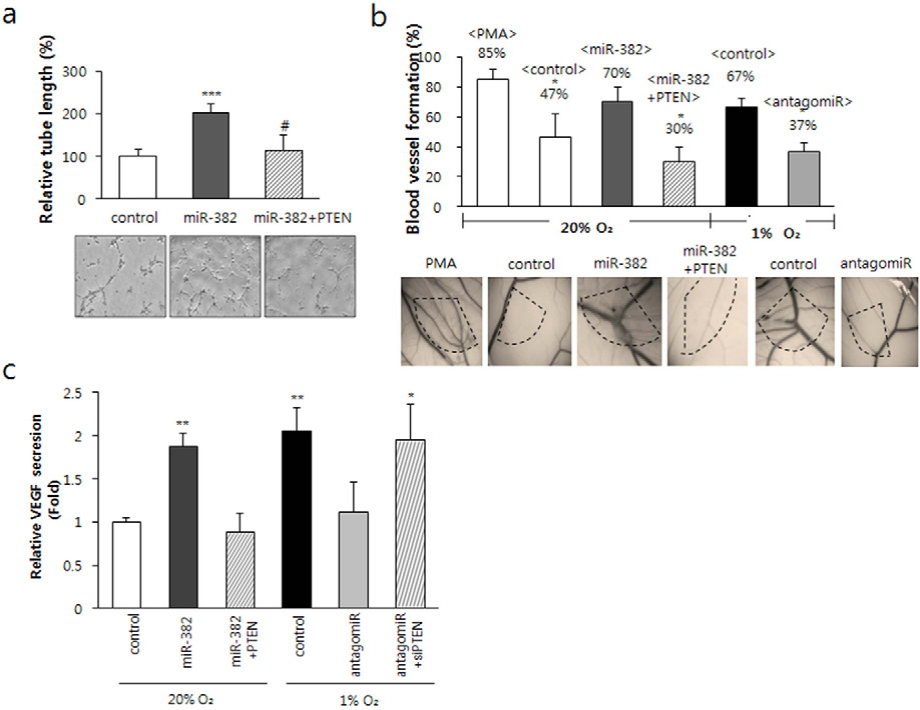
miR-382-induced angiogenesis was inhibited by PTEN. (**a**) CM was obtained from MKN1 cells transfected with PTEN full-length plasmid and/or miR-382, and tube formation assays were performed for 24 h using BAECs. Significance, ***P < 0.0001 versus the control, ^#^*P* < 0.001 versus the miR-382 group. (**b**) CAM assay was performed (as described in ‘Materials and Methods’ section) using the CM used in (a) and CM obtained from MKN1 cells transfected with antagomiR-382. Percentages of eggs positive for new vessel formation among all eggs tested were calculated; values are inserted above the bars in the figure. *P < 0.01 versus PMA control or hypoxic control. (**c**) VEGF secretion into media from cells transfected with miR-382 under normoxia or with antagomiR-382 under hypoxia for 24 h, with PTEN full-length plasmid and/or miR-382 oligomers under normoxia for 24 h were detected by ELISA. Data are presented as means ± SDs (*n* = 6). *P < 0.05 or ***P < 0.001 versus normoxic control. ^#^*P* < 0.05 versus miR-382 or antagomiR-382 group.

To confirm the role of PTEN in miR-382-induced angiogenesis, we utilized CM from miR-382 overexpressing cells for *in vitro* and *in vivo* angiogenesis assays. As shown in Figure 6a, PTEN inhibited the miR-382-induced tube formation of vascular ECs. The CAM assay also demonstrated that PTEN significantly suppressed miR-382-induced *in vivo* angiogenesis to a degree similar to that induced by antagomiR-382, under hypoxic conditions (Figure 6b), indicating that miR-382 induces angiogenesis by targeting PTEN under hypoxic conditions. To investigate whether VEGF, a key angiogenic factor in hypoxia, is required for miR-382-induced angiogenesis, we checked secreted VEGF levels. Hypoxia and miR-382 were found to induce VEGF to similar extents, whereas antagomiR-382 inhibited hypoxia-induced VEGF secretion (Figure 6c). As expected, PTEN siRNA recovered the antagomiR-382-induced inhibition of VEGF secretion under hypoxic conditions (Figure 6c). Furthermore, the overexpression of PTEN significantly reduced miR-382-induced VEGF secretion (Figure 6c). These results suggest that miR-382 induced by hypoxia regulates PTEN and that VEGF is a real angiogenic mediator of miR-382-induced angiogenesis under hypoxia.

These results suggest that miR-382 is a crucial regulator of tumor angiogenesis via VEGF under hypoxic conditions, and it achieves this by targeting PTEN.

## DISCUSSION

This study shows that miR-382 upregulation by hypoxia participates in hypoxia-induced angiogenesis, and in particular, miR-382 directly targets PTEN, a tumor suppressor, and stimulates the AKT/mTOR signaling pathway. To identify novel angiogenic miRs in hypoxic tumor microenvironment, we used a microRNA microarray, three algorithmic programs, a 3′-UTR-luciferase assay, *in vitro* and *in vivo* angiogenic assays, and an animal tumor xenograft model. To modulate the expression of a novel angiogenic miR, miR-382, we used either a chemically modified antisense oligonucleotide (antagomiR) or synthetic miR-382 mimics.

The stimulation of angiogenesis is a characteristic feature of hypoxia (24). Angiogenic molecules, such as VEGF, bFGF, angiopoietin 2 and their receptors are upregulated by hypoxia (25). Recent studies have revealed that microRNAs regulate angiogenesis in ECs (now referred to as angiomiRs) (16). miR-210 is the most dominant angiomiR (26) and is induced by hypoxia (hypoxamiR) in a broad spectrum of cancer types (27). In the present study, several miRs were found to be upregulated by hypoxia, including miR-210 (Figure 1) in gastric cancer cells (MKN1). However, the scarcity of miR-382 in angiogenesis research made us select miR-382 for our experiments. Given the role of hypoxia in angiogenesis, it might be expected that hypoxia-induced miR-382 plays a role in this event. Under hypoxic conditions, many miRs are up- or downregulated to tune the vascular homeostasis to preferred angiogenic processes. Hypoxia-induced miRs, such as Let-7 and miR103–104 are induced by HIF-1α and target argonaute 1, which in turn increases VEGF mRNA expression and tumor xenograft growth (28). However, some miRs such as miR-503 downregulated by HIF-1α inhibit tumor angiogenesis by targeting FGF2 and VEGF (29). Additionally, microRNAs also activate HIF-1α. In fact, miR-21 induced the expression of HIF-1α, VEGF and also activated the AKT and ERK pathways (30). In the present study, miR-382 was found to be associated with tumor growth by increasing angiogenesis *in vivo* and *in vitro* and by decreasing the expression of PTEN (a tumor suppressor). Accordingly, elevated miR-382 expression may be a tumor-specific marker in different cancer cells.

Evidence indicates the involvement of microRNAs in the pathogenesis of human cancer. For example, they have been reported to have a causative role in tumorigenesis and to act as oncogenes or tumor suppressor genes (TSG) in a target specific manner (31). PTEN is a multifunctional TSG and its expression is frequently lost in various cancers. Moreover, the PTEN/AKT/mTOR pathway is involved in the regulation of the cell cycle, proliferation, apoptosis and metastasis during tumorigenesis (32). In the present study, miR-382 was found to target PTEN directly by binding to its 3′-UTR region and to function as an oncogene and an angiogenic inducer under hypoxic conditions. PTEN is also a direct target of miR-106b∼25 cluster in prostate cancer (33), of miR-155 in hepatocellular carcinoma (34), and of miR-214 in ovarian cancer (35). These reports indicated that various miRNAs contribute in the attenuation of PTEN expression in a cancer type dependent manner. Also known as proto-oncogenes or TSGs, microRNAs can regulate angiogenic molecules and/or their signaling pathways. Here, we suggest that VEGF is involved in miR-382-induced angiogenesis because VEGF secretion by hypoxia was reduced by antagomiR-382 and recovered by PTEN siRNA, and because PTEN overexpression suppressed miR-382-induced VEGF secretion (Figure 6c and d). These findings suggest that increased VEGF by miR-382 might be mediated by increased HIF-1α synthesis. Furthermore, because increased HIF-1α synthesis is mediated by the AKT/mTOR signaling pathway (36), an increase in the activity of this pathway by miR-382 might crucially increase angiogenesis by inducing VEGF via HIF-1α (Figure 5).

The stabilization of HIF-1α by hypoxia enhances miR-382 transcription by HIF-1 binding to the HRE site of miR-382 promoter (Figure 2). There are two HRE sites within 4 kb upstream of the miR-382 transcription starting site. In the present study, knockdown of HIF-1α by siRNA clearly diminished miR-382 promoter reporter activity induced by hypoxia. The famous hypoxamiR, miR-210, targets various genes, including BNIP-3, an anti-apoptotic gene that increases cancer cell proliferation and survival (37). Furthermore, it has been well documented that HIF-1 regulates miR-210 expression (38). Thus, it appears that the increase of HIF-1 activity might be a first step in miR-382-induced angiogenesis and oncogenesis.

miR-382 is one of the microRNAs in chromosome 14q32 locus and has been reported to be induced in the brain of the Rett syndrome model (39) and in the olfactory neuroepithelium of schizophrenia patients (40). miR-382 also target superoxide dismutase 2 (SOD2) to mediate TGF-β1-induced epithelial-mesenchymal transition (EMT) (41), which results in the acquisition of cell invasion and drug resistance, and reversion to cancer stem cells (42). Loss of epithelial characteristics is also induced by miR-382 in renal interstitial fibrosis (43). MicroRNAs in chromosome 14q32 locus are downregulated in osteosarcomas, suggesting that these 14q32 microRNAs, including miR-382, potentially target the cMyc transcript and that the microRNAs-cMyc gene network is deregulated during the pathogenesis of osteosarcoma (44,45). The involvement of miR-382 has also been reported during leukemogenesis (46).

In solid tumor, in tumor cells far from the vessel, around 35 μm, *p*O_2_ was almost 7.6 mmHg (47), which correspond to what we used in this study (1% O_2)_. Therefore, cells inside a tumor mass larger than 1 mm^3^ can experience hypoxic conditions. From a recent meta-analysis, increased HIF-1α and aberrant expression of PTEN can be used to predict the prognosis of gastric cancer (48). In addition, HIF-1 level presents a strong correlation with poor prognosis of gastric cancer (49). As HIF-1α is also synthesized by PI3K/AKT/mTOR pathway in hypoxia-independent mechanism (36), inhibition of miR382 can increase PTEN level, which inhibits the PI3K/AKT/mTOR pathway and in turn decrease HIF-1α. Therefore, taken together with our results and recent findings from other groups, chemotherapeutic efficacy for gastric cancer can be increased by the inhibition of the AKT/mTOR pathway (50), and cancer progression and metastasis through increased angiogenesis through miR-382 (Figures 3 and 6) can be inhibited by PTEN or antagomiR-382 in hypoxic gastric cancer.

As HIF-1α is a critical factor for drug resistance, EMT, metastasis and maintenance of cancer stem cell properties (51), miR-382 may have a role in the development of many diseases mentioned above, via HIF-1α or via targeting PTEN tumor suppressor. Increasing reports indicate that microRNAs in the chromosomal region 14q32 may be associated with cancer progression and multi-drug resistance, but the role of individual miRs in 14q32 and miR-382 in cancer progression or other disease pathogenesis should be investigated further. However, from our findings and other reports, it appears that miR-382 is a strong oncomiR and hypoxamiR candidate, and that it is a potential therapeutic target and prognostic indicator in various types of cancer, especially in gastric cancer.

## SUPPLEMENTARY DATA

Supplementary Data are available at NAR Online.

SUPPORTING INFORMATION
