# Supplementary material for: MicroRNA-382 induced by HIF-1α is an angiogenic miR targeting the tumor suppressor phosphatase and tensin homolog
Source: Nucleic Acids Res. 2014 Jun 9;42(12):8062–72. doi: 10.1093/nar/gku515 (PMC4081109; doi:10.1093/nar/gku515)
Supplement: SUPPORTING INFORMATION [file supp_42_12_8062__index.html]

MicroRNA-382 induced by HIF-1α is an angiogenic miR targeting the tumor suppressor phosphatase and tensin homolog — SUPPORTING INFORMATION 

# MicroRNA-382 induced by HIF-1α is an angiogenic miR targeting the tumor suppressor phosphatase and tensin homolog

## SUPPORTING INFORMATION

**Files in this Data Supplement:**

- Supplemental Figures 1
- Supplemental Figures 2
