# Supplementary figures and images for: MicroRNA-382 induced by HIF-1α is an angiogenic miR targeting the tumor suppressor phosphatase and tensin homolog
Source: Nucleic Acids Res. 2014 Jun 9;42(12):8062–72. doi: 10.1093/nar/gku515 (PMC4081109; doi:10.1093/nar/gku515)

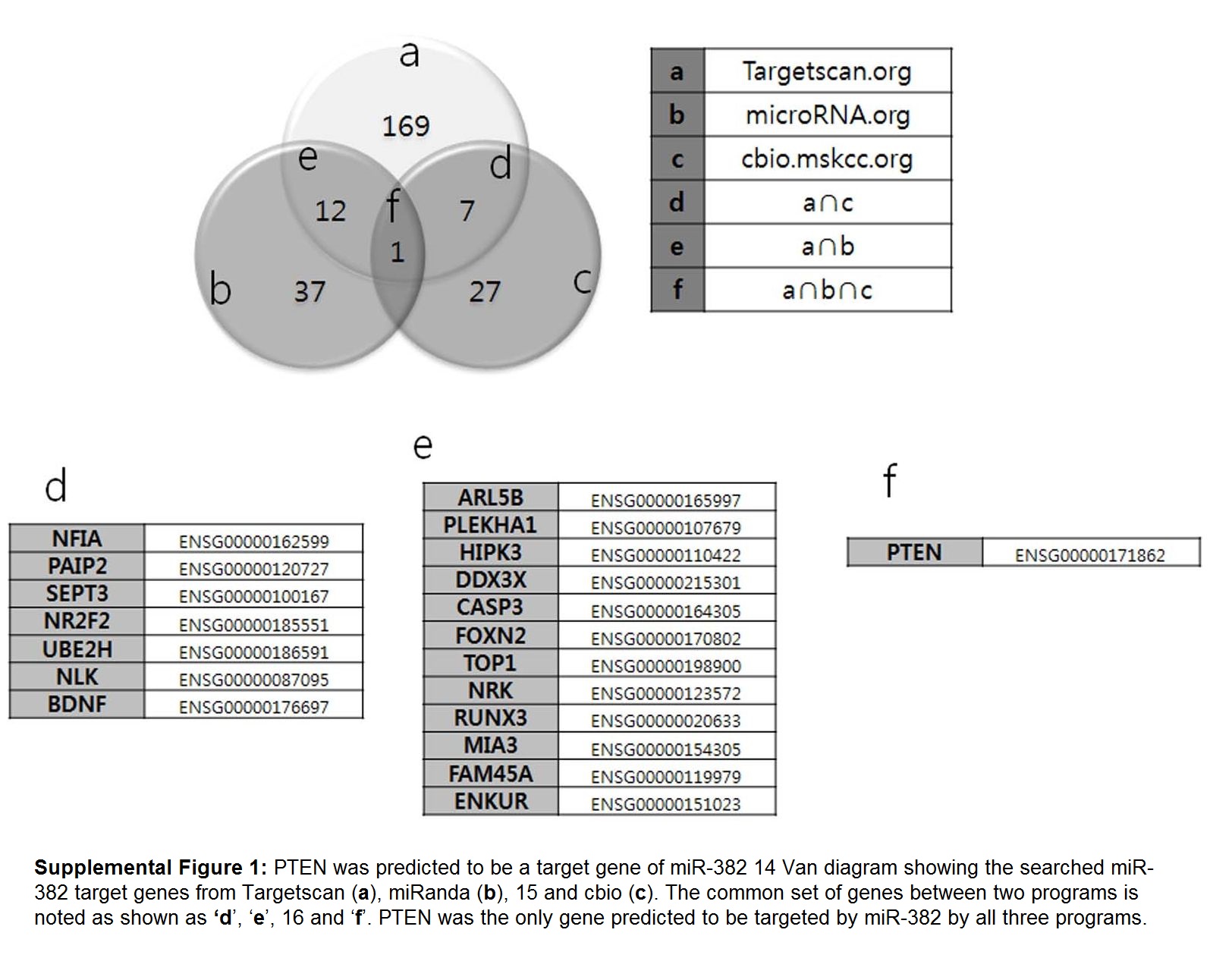

Supplement: SUPPORTING INFORMATION [file supp_gku515_nar-00328-x-2014-File009.jpg]

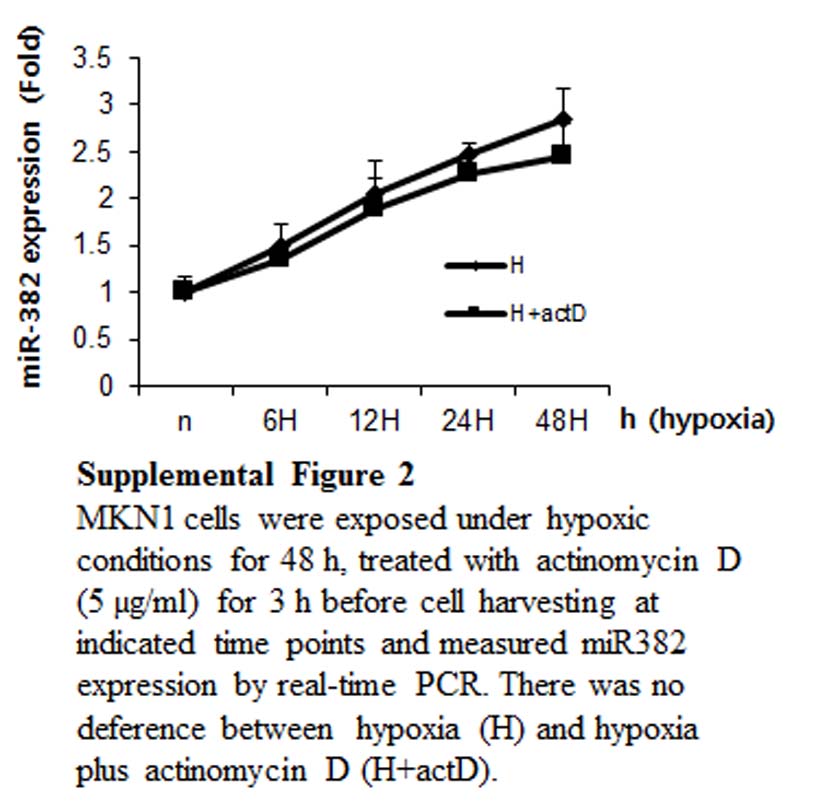

Supplement: SUPPORTING INFORMATION [file supp_gku515_nar-00328-x-2014-File010.jpg]
